# Phenotypic Effects of Salt and Heat Stress over Three Generations in *Arabidopsis thaliana*

**DOI:** 10.1371/journal.pone.0080819

**Published:** 2013-11-14

**Authors:** Léonie Suter, Alex Widmer

**Affiliations:** ETH Zürich, Institute of Integrative Biology, Zürich, Switzerland; National Taiwan University, Taiwan

## Abstract

Current and predicted environmental change will force many organisms to adapt to novel conditions, especially sessile organisms such as plants. It is therefore important to better understand how plants react to environmental stress and to what extent genotypes differ in such responses. It has been proposed that adaptation to novel conditions could be facilitated by heritable epigenetic changes induced by environmental stress, independent of genetic variation. Here we assessed phenotypic effects of heat and salt stress within and across three generations using four highly inbred *Arabidopsis thaliana* genotypes (Col, Cvi, Ler and Sha). Salt stress generally decreased fitness, but genotypes were differently affected, suggesting that susceptibility of *A. thaliana* to salt stress varies among genotypes. Heat stress at an early rosette stage had less detrimental effects but accelerated flowering in three out of four accessions. Additionally, we found three different modes of transgenerational effects on phenotypes, all harboring the potential of being adaptive: heat stress in previous generations induced faster rosette growth in Sha, both under heat and control conditions, resembling a tracking response, while in Cvi, the phenotypic variance of several traits increased, resembling diversified bet-hedging. Salt stress experienced in earlier generations altered plant architecture of Sha under salt but not control conditions, similar to transgenerational phenotypic plasticity. However, transgenerational phenotypic effects depended on the type of stress as well as on genotype, suggesting that such effects may not be a general response leading to adaptation to novel environmental conditions in *A. thaliana*.

## Introduction

In the face of climate change, many organisms may be forced to adapt to novel, potentially challenging environmental conditions that may often exceed their typical range of reaction [1]. Alternatively, organisms may have to migrate and track suitable habitats to escape extinction. Changing environmental conditions, such as increasing temperature, may thus have important consequences for species diversity in natural ecosystems [2], but also for crop production, since increasing temperatures can significantly decrease yield in many crop species [3]. Another major environmental factor affecting agriculture and natural vegetation is soil salinity, to date a problem in more than 100 countries worldwide [4]. To assess the potential of species to cope with such environmental challenges it is on the one hand important to know the species’ norms of reaction, i.e. the phenotypic response within one generation, and on the other hand the species’ ability to adapt over a number of generations to changing environmental conditions.

Molecular and phenotypic effects of heat or salt stress are well studied in *Arabidopsis thaliana*. Heat stress is known to affect gene expression [5,6] and many studies have assessed the roles of heat shock proteins in stress response [7,8]. Generally, heat stress is expected to have negative fitness consequences [9-11], and the timing of heat stress may be most important: heat stress during seed production can have highly detrimental fitness effects [9,10], whereas similar conditions at vegetative plant stages may have much milder fitness consequences [12]. Similarly, knowledge about molecular changes induced by salt stress is increasing [13,14] and a number of studies have examined the phenotypic consequences of saline conditions in *A. thaliana* [15-17]. Compared to other species, *A. thaliana* is highly sensitive to saline conditions [18], although remarkable differences between *A. thaliana* accessions have been reported [16,17].

While the consequences of environmental stress on phenotypes and molecular processes within generations are relatively well explored in *A. thaliana*, much less is known about transgenerational effects. A number of studies have suggested that later generations can be influenced by environmental conditions experienced by preceding generations [9,19-21]. However, the underlying mechanisms often remain elusive. 

If genetic variability can be excluded, epigenetic inheritance is one candidate cause for environmentally induced transgenerational effects. Epigenetic inheritance encompasses anything heritable apart from DNA, although usually, three main mechanisms are distinguished: DNA methylation, histone modifications and inheritance of small RNA molecules [22-24]. Importantly, epigenetic patterns are not only heritable, but can also directly be influenced by the environment. For example, in apomictic dandelion, Verhoeven et al. [25] found altered DNA methylation upon environmental stress exposure, which was mostly heritable to the next generation. However, apart from epigenetic effects, environmental stress may also destabilize the genome and lead to an increase of genomic mutations [26], e.g. through mobilization of transposable elements in response to heat stress [27] or an increase of homologous recombination upon salt stress [28]. With phenotypic data alone it is difficult to disentangle genetic and epigenetic transgenerational effects of environmental stress. However, a relatively high number of genetically identical, independent replications and correlations between parent and offspring phenotypes may reveal whether observed transgenerational phenotypic effects are genetically or epigenetically inherited. 

If genetic diversity can be excluded, heritable epigenetic variability could significantly contribute to phenotypic diversity and thus to the adaptive potential of a species [29]. Furthermore, epigenetic modifications can evolve much faster than genetic mutations and may be especially relevant when rapid adaptation is required [30,31]. Additionally, resetting of epigenetic marks would allow an organism to react to variable conditions [26,32]. The resulting transgenerational phenotypic effects of environmental stress could in principle fall into three categories: tracking, phenotypic plasticity and bet-hedging [33]. Tracking is typically understood as a change in mean of a phenotypic trait due to altered natural selection in a novel environment, resulting in a shift of allele frequencies within a population [33]. In the absence of genetic variability and selection on existing genetic variation, a similar effect could be observed if environmental stress induces heritable epigenetic alterations that lead to a shift in mean phenotype over generations. Phenotypic plasticity could similarly result from transgenerational epigenetic inheritance triggered by environmental stress experienced in preceding generations, but one would then expect a plastic interaction with the current environment, i.e. a phenotypic shift due to ancestral treatment could be observed in one present environment, but not in another. Phenotypic plasticity is advantageous when environments are variable, but can be predicted reliably within an organism’s lifetime [34,35]. However, if environments cannot be predicted adequately, e.g. due to rapid fluctuations or missing detectability from organisms, diversified bet-hedging can be adaptive [33,36]. Diversified bet-hedging refers to a strategy where one genotype produces phenotypically highly variable offspring, independent of environmental conditions. This way the risk is spread among offspring and a fraction of the offspring may express an appropriate phenotype, decreasing the variance in offspring fitness over different environments [33,37]. 

To what extent plants can express such transgenerational phenomena and what environmental conditions are necessary to trigger them, is presently unclear. A major problem is that different studies often apply environmental stresses differently, which may prevent meaningful comparisons among studies. For example, heat shock response has been studied at temperatures ranging from 38 °C to over 50 °C in *A. thaliana* [38], and it is not surprising that results of such studies are often incongruent. Additionally, the applied environmental stresses are often not mimicking natural conditions, such as in the case of sudden shifts in temperature to achieve maximal heat shock [39,40]. Not surprisingly, results of such studies can differ significantly from studies applying more realistic scenarios, such as for example gradual temperature increases [6]. This raises the question as to whether results from unrealistic experimental designs may uncover adaptive responses or instead report artifacts induced by unnatural stress conditions. 

The goal of the present study was to assess the phenotypic responses of *A. thaliana* to realistic heat and salt stress conditions. To get an impression of how consistent plants responded to stresses within and across generations, we tested four different accessions, each with a relatively high number of replications over three generations of stress or control treatment. Specifically, we addressed the following questions: How does *A. thaliana* react to (realistic) environmental stress conditions and are there differences between genotypes? Does ancestral stress treatment influence phenotypes in stress or control treatments over three generations, and if yes, what are the modes of response (tracking, phenotypic plasticity, bet-hedging)? If three generations of stress lead to transgenerational phenotypic changes, are these changes adaptive?

## Materials and Methods

### Plant material and growth conditions

Four widely studied *Arabidopsis thaliana* accessions were used: Columbia (Col-0), Landsberg *erecta* (L*er*-0), Cape Verde islands (Cvi-0) and Shahdara (Sha-0; hereafter referred to as Col, Ler, Cvi and Sha, respectively). One plant per accession was grown in generation 0 (G0) to minimize genetic diversity and all plants grown in generation 1 (G1) were descendants of these G0 plants. 

Plants were grown in individual 7 x 7 x 8 cm pots filled with Biouniversalerde (Oekohum GmbH, Herrenhof, Switzerland), an all-purpose soil without peat. Pots were randomly arranged on 28-pot-trays (G0, G1) or 24-pot-trays (generations 2 and 3 (G2, G3)) that were randomized three times per week to avoid position effects. Randomization of trays was stopped upon maturation of siliques. Approximately five seeds were sown per pot to ensure successful germination. Throughout the experiment, plants were grown in climate chambers (Kälte 3000, Landquart, Switzerland) to equalize growth conditions over generations. Seeds were stratified at 4 °C in the dark for five days to break seed dormancy. After stratification, all plants were moved to climate chambers, and this day was counted as day 0 of the experiment for each generation. About one week after germination, seedlings were thinned to one plant per pot. Upon flowering, individual pots were packed in Arabisifter floral sleeves (Lehle Seeds, Round Rock, Texas, USA) to avoid cross-pollination between neighboring plants and to harvest seeds. Plants were watered once per week with tap water containing Solbac (Andermatt Biocontrol AG, Grossdietwil, Switzerland), diluted according to manufacturers instructions. Watering was stopped two weeks before seed harvest. 

In G0, all plants were grown under control conditions (10 kLux light for 16 h, dark for 8 h; 22 °C/18 °C day/night temperatures; 50 %/60 % relative humidity at day/night). In G1 – G3, plants were either grown in control conditions or in one of two stress conditions. 

### Heat stress

To find the best setting for heat treatments, a pre-experiment was conducted. Col plants were heat treated at 32 °C, 36 °C or 40 °C for 1, 2 or 3 days, either 7 or 14 days after germination. Plants that were heat treated for more than 1 day were allowed to recover for 2 - 3 days between heat treatments. Overall, 18 different heat treatments with eight replications each were tested (for details see Table S1). As even the most extreme heat treatment (three days at 40 °C) had no major impact on plants fitness (data not shown) we decided to expose plants to heat stress at 40 °C for three successive days, starting at day 12 of the experiment. For this heat treatment, temperatures were gradually increased during the day over 7 h to reach 40 °C. This temperature was kept for 2 h followed by a gradual decrease over 7 h back to 18 °C. Light and relative humidity were identical to control conditions, as were night conditions. After three days of heat stress, plants were continued to be grown under control conditions. 

### Salt stress

A pre-experiment was conducted to identify suitable salt concentrations to apply salt stress. Eight replications per treatment of genotype Col were watered for the first four weeks with NaCl-solution with concentrations of 25 mM, 50 mM, 100 mM or 150 mM. 25 mM NaCl had no major effect on plant fitness, while none of the plants watered with 100 mM or 150 mM NaCl-solution survived to set seeds. Plants treated with 50 mM NaCl-solution showed somewhat reduced fitness, but still survived and produced seeds (data not shown). Therefore, we decided to water plants in the salt treatment with 50 mM NaCl for the first four weeks of each generation, followed by normal watering thereafter. In G2 of the full experiment, only one plant of genotype Cvi survived this salt treatment, therefore this genotype had to be excluded from the salt experiment.

### Experimental design

**Figure 1:**
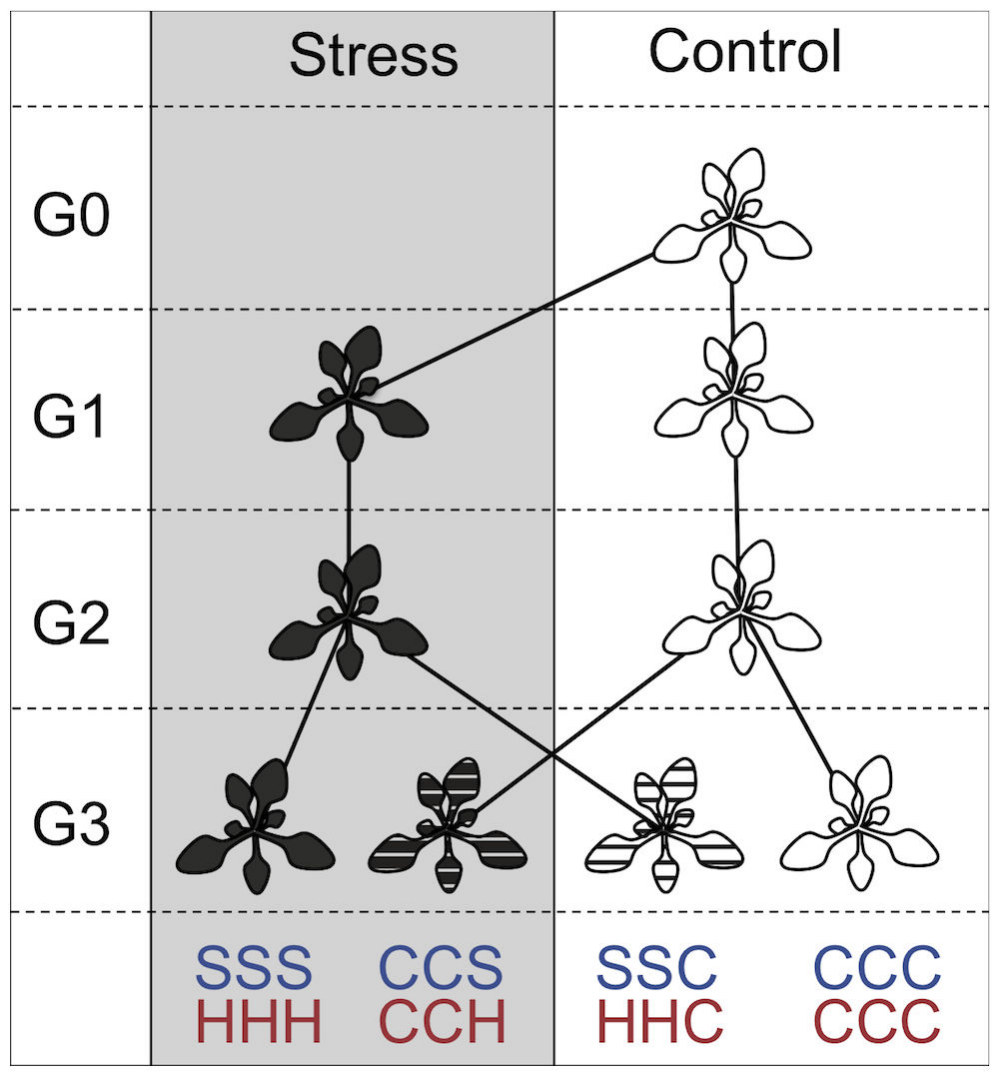
Setup of the experiment. G0 = generation 0, G1 = generation 1 etc. In blue, SSS refers to three generations of salt treatment, CSS indicates that the first generation was grown under control conditions followed by two generations in stress treatment etc., while in red HHH refers to three generations of heat treatment, etc. In G0, only one individual was grown, whereas in G1 to G3, 25 individuals per treatment were grown. Plants were propagated by single seed descent, and in case of mortality, missing plants were restocked by duplicating other lines of the same treatment.

In G1, 25 replications per accession were grown either under heat, salt or control conditions, and propagated by single seed descent to the next generation, G2, where plants were grown under the same conditions. In most accession × treatment combinations, not all 25 replicated plants of G1 survived to produce seeds. Missing lines were replaced in generation 2 (G2) by taking seeds of randomly chosen G1 lines such that again 25 plants per accession and treatment were grown in G2. The same procedure was applied for G3. Care was taken to maintain as many independent replications as possible in G3 (see Table S2). Due to space restrictions, it was not possible to grow salt and heat treated plants at the same time after G1. Consequently, control plants were also grown separately (together with each stress treatment) as from G2. Offspring of G2 plants were grown both under stress and under control conditions. Therefore, in G3, plants with four different histories were grown (see Figure 1): stress treatment in G1 – G3 (hereafter abbreviated SSS for salt stress, HHH for heat stress), control treatment in G1 and G2 followed by stress treatment in G3 (CCS, CCH), stress treatment in G1 and G2 and followed by control treatment in G3 (SSC, HHC) and control treatment in G1 – G3 (CCC). 

To estimate growth, phenological parameters, fitness and plant architecture, a large number of phenotypic traits, including flowering time, number of rosette leaves, and total number of siliques, were measured in G3 (Table S3), with a subset of traits also measured in G1 and G2. Some of these phenotypic traits best describe variation in life history traits among genotypes [41].

### Data analysis

All statistical analyses were conducted using R [42]. As heat and salt treatments had very different effects on phenotypic traits, data were analyzed separately for heat and salt treatments. Means and standard deviations of all phenotypic traits computed separately for each genotype and treatment combination (HHH, CCH, etc.) are presented in Table S4 and S5. 

**Figure 2:**
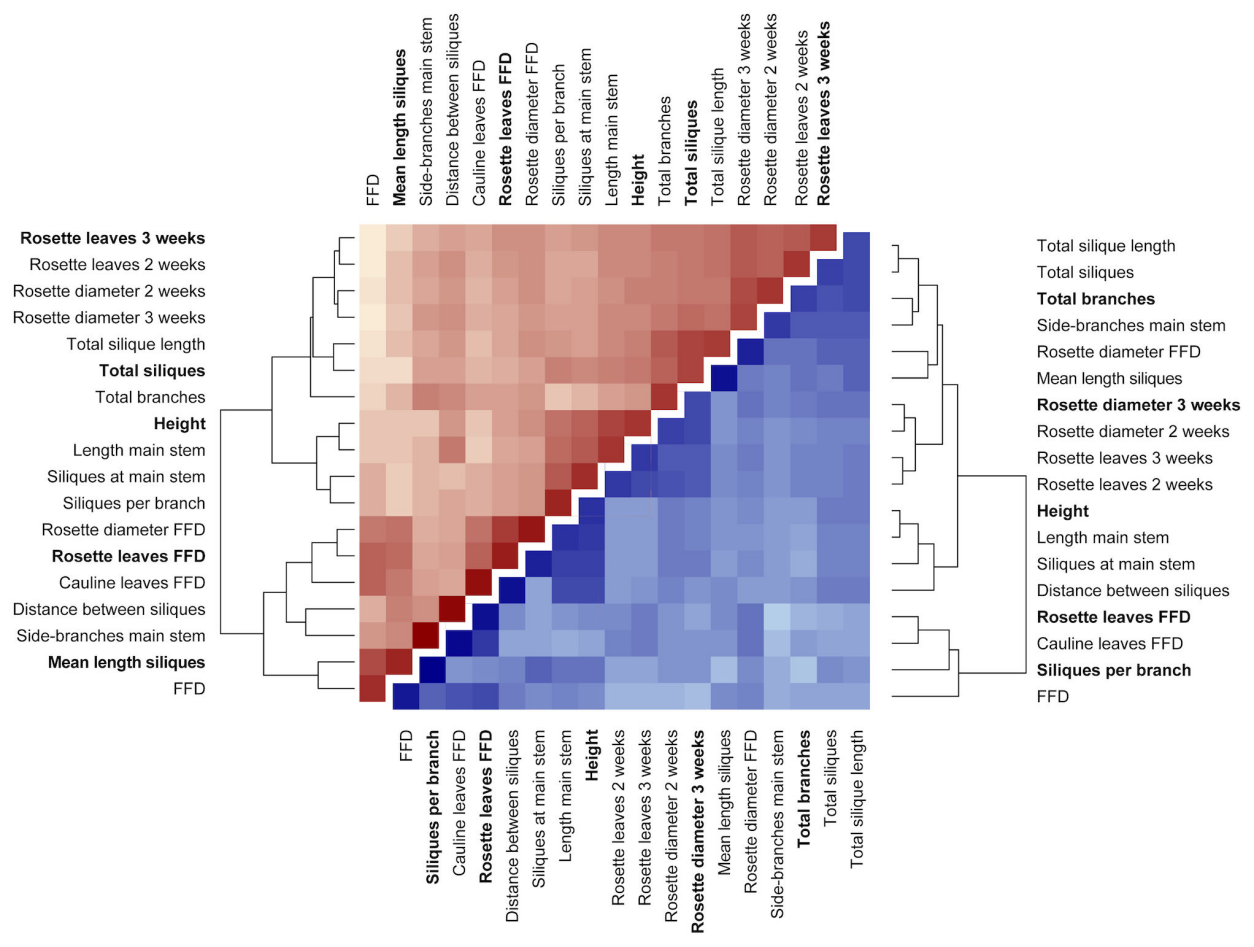
Correlation analysis to select a subset of independent phenotypic traits. The heat map shows pairwise Pearson correlations between phenotypic traits (darker colors denote stronger correlations), with data from the heat stress experiments above the diagonal in red and data from the salt stress experiment below the diagonal in blue. Phenotypic traits written in bold letters were selected for statistical analyses.

*Selection of phenotypic traits* – Many of the measured traits strongly correlated with each other, e.g. diameter and rosette leaves after two and after three weeks (Figure 2). We were primarily interested in how many independent, i.e. non-correlated traits were affected by the applied stress treatment, as they may also be independent from each other on a molecular level. To select non-correlated traits, pairwise Pearson correlations were computed and plotted to a heatmap, where traits were reordered according to their similarities determined with a dendrogram. From the resulting clusters of traits, five traits that were both responsive to the applied stress and not correlated with each other were selected both for salt and heat stress (Figure 2). 

**Figure 3:**
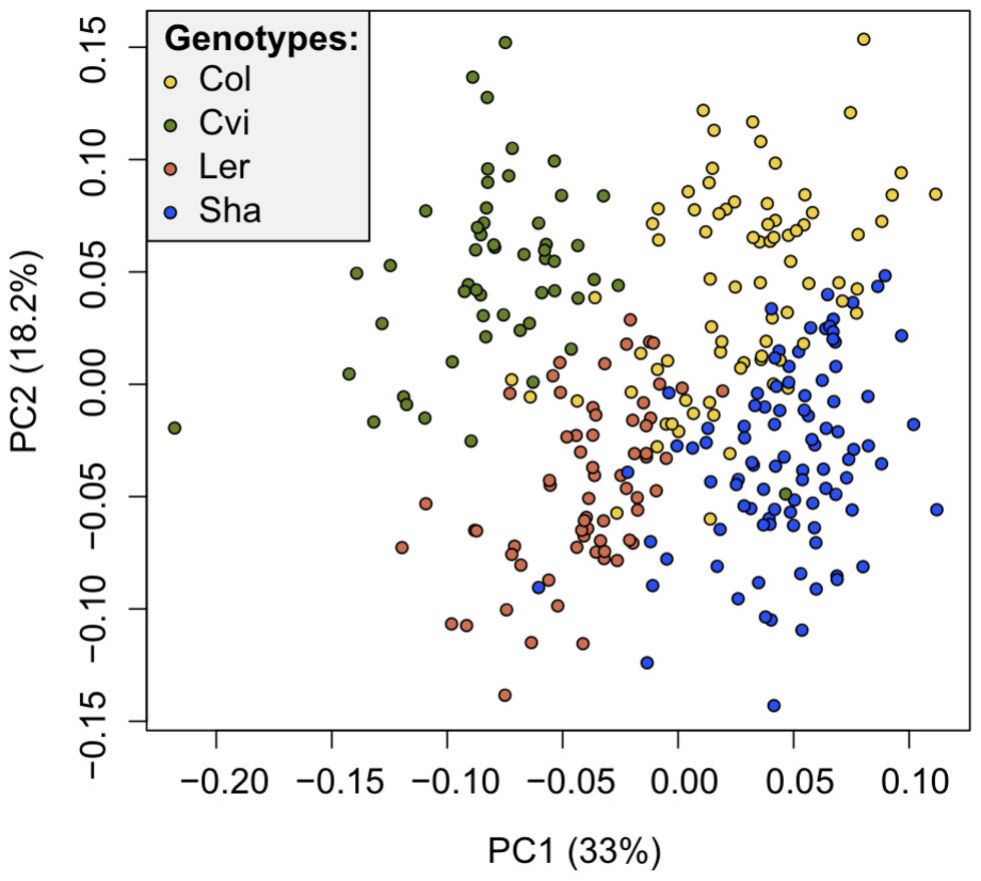
Principal component analysis of data from the heat experiment. Colors denote different genotypes, indicating their distinct phenotypes.

*Effect of genotypes*- initially we performed a linear mixed effect model with genotype, previous treatments (G1 and G2) and G3 treatments as well as their pairwise interactions as fixed and tray as random factors. As genotype had a dominant effect on all measured traits (Tables S6 and S7), principal component analyses with the full set of phenotypic traits were performed to further assess the effect of genotypes. Both for the heat and the salt data the genotypes clearly clustered separately (Figure 3 and Figure S1), thus all further analyses were performed for individual genotypes. 

*Effect of stress treatments* – Linear mixed effect models with previous treatment (G1 and G2) and G3 treatment as well as their interactions as fixed and tray as random factor were computed separately for each genotype. P-values were adjusted for multiple testing separately for each genotype following Benjamini and Hochberg [43]. Where significant effects of previous (G1 and G2) treatments or interactions between previous and G3 treatments were observed, effects of previous treatments were calculated separately for each G3 treatment. To assess the effect of previous treatments on the variance of phenotypic traits, F tests were performed separately for each G3 treatment. As trays had a significant effect on several traits, we first estimated tray effects using a linear model and used the residuals of these tests for all further calculations. P-values were adjusted for multiple testing following Benjamini and Hochberg [43].To test whether increased variance could be explained by inheritance of extreme phenotypes in G2, a Spearman correlation for a subset of traits was calculated between G3 plants and their progenitors grown in G2. To assess whether increased variance may allow for different life history strategies (bet-hedging), five Cvi plants with the highest fitness (number of siliques) in treatments HHH, HHC, CCH and CCC were identified. Plants with the same ancestral treatment were compared with a linear model to test whether traits differed between G3 treatments (e.g. HHH vs. HHC). Similarly, the five plants with the lowest number of siliques were compared, as well as all Cvi plants independent of silique numbers. 

## Results

### Phenotypic effects of environmental stress in generation *3*

**Figure 4:**
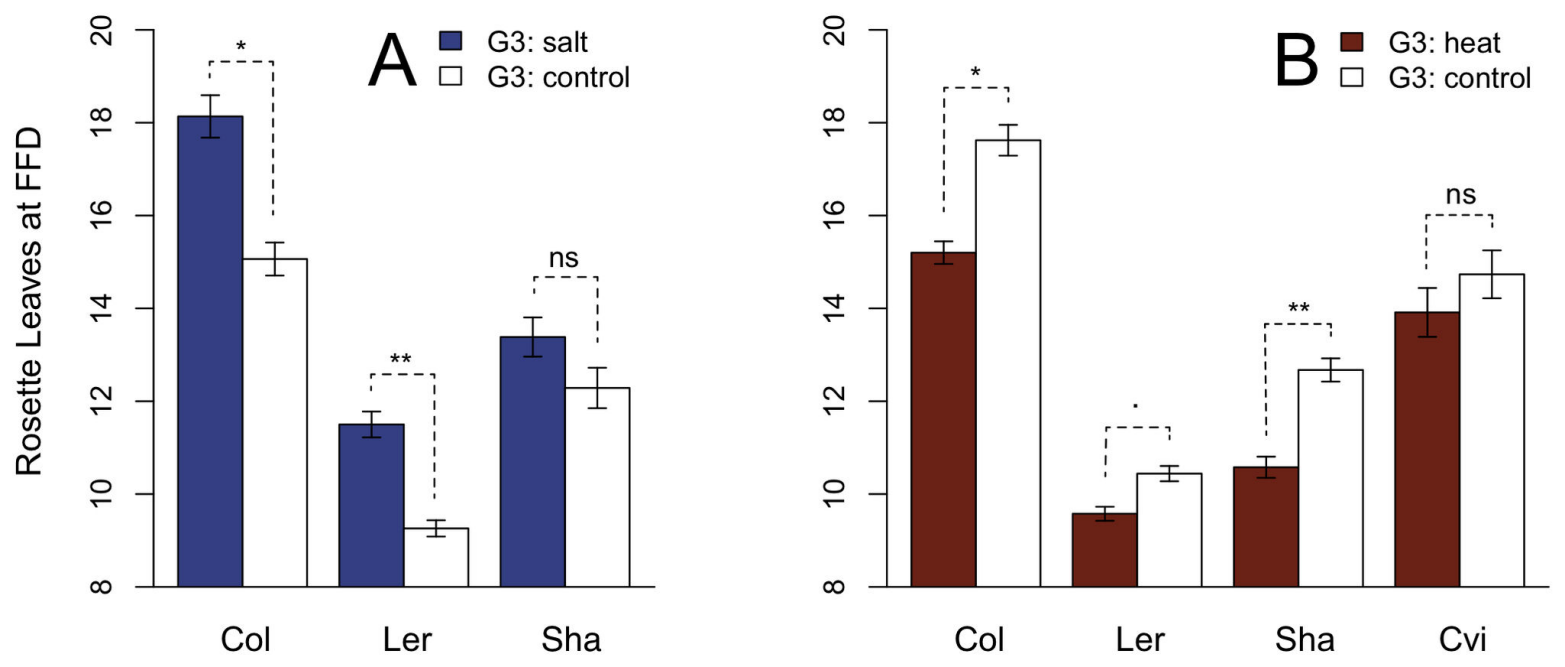
Effect of stress treatments on rosette leaves at FFD. A: Col, Ler and Sha grown in G3 under salt (blue) and control (white) conditions. B: Col, Ler, Sha and Cvi grown in G3 under heat (red) and control (white) conditions. ** P-value < 0.01; * P-value < 0.05, · P value < 0.1, ns: P-value not significant. P-values were adjusted for multiple testing according to Benjamini and Hochberg [43].

*Salt treatment* – Exposure to salt stress generally reduced fitness in all genotypes when compared to plants grown under control conditions (Table 1). Plants were shorter and had reduced numbers of branches in all genotypes. Additionally, in genotypes Col and Ler, the number of rosette leaves at FFD, a trait that indicates at what physiological age the transition from vegetative to reproductive stage takes place, was increased under salt conditions, indicating a delay of flowering when compared to control plants (Figure 4A, Table 2). Overall, the response of genotype Sha to salt treatment appeared weaker than in the other genotypes, with no significant delay in flowering and a relatively small decrease in total branches (Figure 4A, Table 2). 

**Table 1:** Effects of G3 and G1G2 salt treatments and interactions on phenotypic traits were calculated for three genotypes using linear mixed models with trays as random factor.

		G3 treatment			G1G2 treatment		G3 x G1G2		
Genotype	Phenotypic trait	F_dF_	P^b^		F_dF_	P^b^	F_dF_	P^b^	
Col	Rosette diameter 3 weeks	5.476_1,8_	0.095	⋅	0.028_1,73_	0.868	1.678_1,73_	0.266	
	Rosette leaves FFD	9.266_1,8_	0.032	*	3.907_1,73_	0.069	0.437_1,73_	0.511	
	Height	45.267_1,8_	<0.001	***	2.159_1,72_	0.195	1.278_1,72_	0.262	
	Siliques per branch	2.135_1,8_	0.307		1.463_1,72_	0.307	0.042_1,72_	0.838	
	Total branches	18.425_1,8_	0.005	**	0.074_1,73_	0.967	0.002_1,73_	0.967	
Ler	Rosette diameter 3 weeks	6.565_1,8_	0.067	⋅	0.002_1,82_	0.965	0.448_1,82_	0.673	
	Rosette leaves FFD	29.030_1,8_	0.001	**	0.303_1,82_	0.583	1.863_1,82_	0.235	
	Height	13.021_1,8_	0.014	*	0.001_1,81_	0.971	1.968_1,81_	0.219	
	Siliques per branch	0.312_1,8_	0.897		0.056_1,78_	0.897	0.017_1,78_	0.897	
	Total branches	20.987_1,8_	0.004	**	0.321_1,82_	0.572	0.395_1,82_	0.572	
Sha	Rosette diameter 3 weeks	2.993_1,8_	0.244		0.185_1,84_	0.668	0.209_1,84_	0.668	
	Rosette leaves FFD	1.586_1,8_	0.324		0.197_1,84_	0.659	1.425_1,84_	0.324	
	Height	47.187_1,8_	<0.001	***	4.315_1,84_	0.054	0.386_1,84_	0.536	
	Siliques per branch	0.480_1,8_	0.508		2.564_1,84_	0.151	6.474_1,84_	0.026	*
	Total branches	14.024_1,8_	0.011	*	0.520_1,84_	0.504	0.451_1,84_	0.504	

b P-values were adjusted for multiple testing according to Benjamini and Hochberg [43], separately for each genotype.

*** P-value <0.001, **P-value < 0.01, *P-value < 0.05, ⋅ P-value < 0.1

**Table 2:** Effects of G3 and G1G2 heat treatments including interactions on phenotypic traits separately analyzed for each genotype using linear mixed models with trays as random factor.

		G3 treatment			G1G2 treatment			G3 x G1G2	
Genotype	Phenotypic trait	F_dF_	P^b^		F_dF_	P^b^		F_dF_	P^b^
Col	Rosette leaves 3 weeks	0.446_1,8_	0.698		2.363_1,65_	0.258		0.000_1,65_	1.000
	Rosette leaves FFD	13.546_1,8_	0.012	*	0.673_1,65_	0.553		0.050_1,65_	0.824
	Height	0.117_1,8_	0.741		0.831_1,63_	0.487		1.275_1,63_	0.487
	Total siliques	0.003_1,8_	0.958		0.549_1,65_	0.615		1.404_1,65_	0.481
	Mean length siliques	6.413_1,8_	0.070	⋅	0.117_1,64_	0.827		0.048_1,64_	0.827
Ler	Rosette leaves 3 weeks	0.445_1,8_	0.795		0.068_1,52_	0.795		0.206_1,52_	0.795
	Rosette leaves FFD	5.900_1,8_	0.083	⋅	0.602_1,55_	0.588		0.030_1,55_	0.863
	Height	0.355_1,8_	0.568		0.652_1,55_	0.564		1.192_1,55_	0.559
	Total siliques	0.395_1,8_	0.547		3.611_1,55_	0.125		0.578_1,55_	0.547
	Mean length siliques	0.559_1,8_	0.635		0.004_1,55_	0.949		2.942_1,55_	0.184
Sha	Rosette leaves 3 weeks	4.770_1,8_	0.081	⋅	10.630_1,81_	0.003	**	0.155_1,81_	0.695
	Rosette leaves FFD	14.922_1,8_	0.010	**	1.995_1,82_	0.215		1.314_1,82_	0.255
	Height	0.366_1,8_	0.562		1.736_1,82_	0.383		1.135_1,82_	0.386
	Total siliques	0.111_1,8_	0.977		0.037_1,82_	0.977		0.001_1,82_	0.977
	Mean length siliques	0.171_1,8_	0.690		0.228_1,82_	0.690		0.169_1,82_	0.690
Cvi	Rosette leaves 3 weeks	1.657_1,6_	0.491		0.323_1,42_	0.764		0.079_1,42_	0.780
	Rosette leaves FFD	1.191_1,6_	0.387		0.763_1,42_	0.387		0.775_1,43_	0.387
	Height	0.631_1,6_	0.792		0.008_1,42_	0.928		0.288_1,43_	0.792
	Total siliques	0.277_1,6_	0.618		0.494_1,42_	0.618		4.187_1,43_	0.094
	Mean length siliques	0.019_1,6_	0.896		0.609_1,42_	0.879		0.088_1,42_	0.896

b P-values were adjusted for multiple testing according to Benjamini and Hochberg [43] separately for each genotype.

** P-value < 0.01, *P-value < 0.05, ⋅ P-value < 0.1

*Heat treatment* – After three days of heat treatment at an early rosette stage genotypes Col, Sha and Ler (trend) had fewer rosette leaves at first flowering day (FFD) when compared to control treatments, while no difference was found in genotype Cv (Table 2, Figure 4B). 

### Phenotypic effects of environmental stress across generations

**Figure 5:**
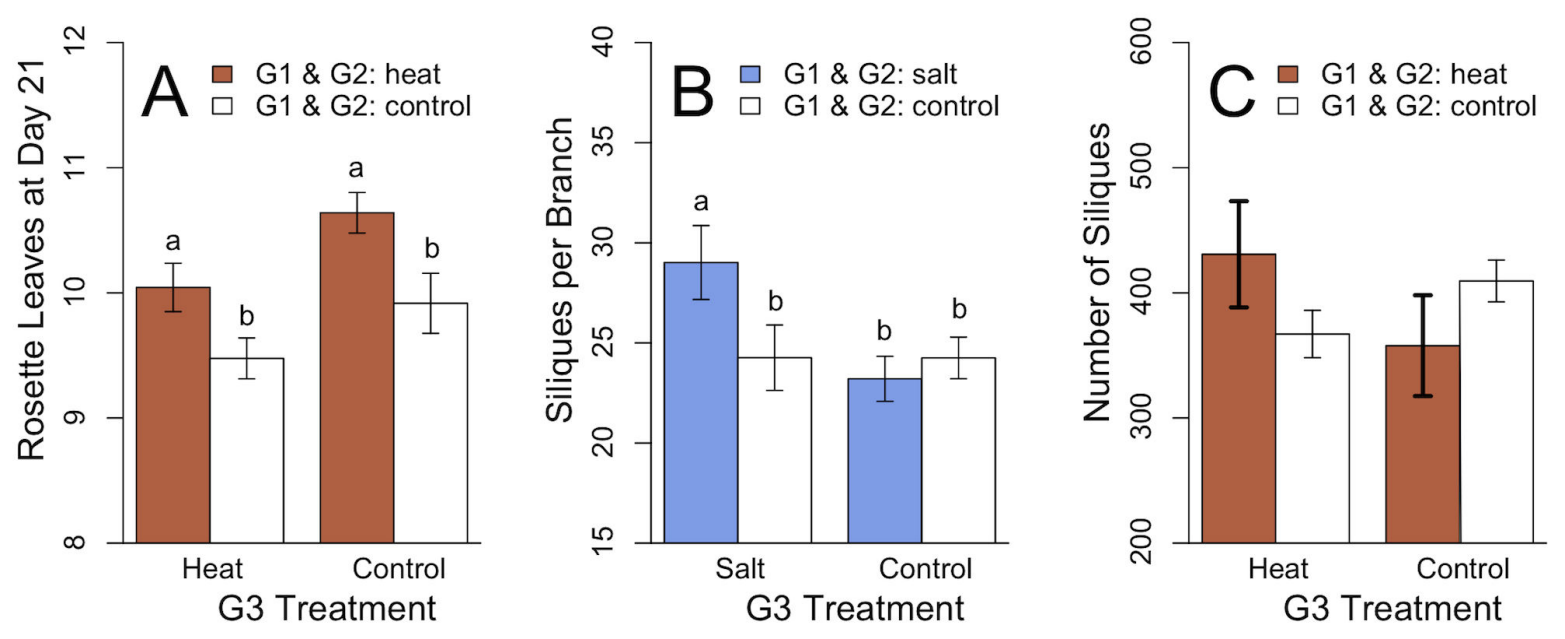
Transgenerational effects of two stress generations displaying three different modes of adaptation to novel environments. A: Tracking: in genotype Sha two generations of ancestral heat treatment (orange) led to more rosette leaves after three weeks in comparison to ancestral control treatment (white). B: Transgenerational phenotypic plasticity: in genotype Sha, two generations of ancestral salt treatment (blue) led to increased number of siliques per branch when compared to ancestral control conditions (white) under G3 salt conditions, but not G3 control conditions. C: Diversified bet-hedging: in genotype Cvi ancestral heat treatment (orange) increased the variance of total siliques in comparison to ancestral control treatment (white).

*Salt treatment –*, We found interactions between previous treatments and G3 treatments for the number of siliques per branch in genotype Sha (Table 1). Specifically, under G3 salt conditions offspring of salt treated plants (SSS) produced more siliques per branch than plants who’s progenitors were grown under control conditions (CCS, F_df_ = 7.191_1,41_, P = 0.011), while no differences were found under G3 control conditions between SSC and CCC (F_df_ = 0.587_1,43_, P = 0.448; Figure 5B). Furthermore, SSS plants grew taller than CCS plants (F_df_ = 4.244_1,41_, P = 0.046), while SSC and CCC plants grew similarly high (F_df_ = 1.071_,43_, P = 0.307), indicating that the plants architecture was altered depending both on previous and G3 treatments.

Previous salt treatments did not affect phenotypic traits measured in genotypes Col and Ler, nor did we find interactions between previous and G3 treatments for these genotypes. 

While ancestral treatment had an effect on the variance of a few traits (Table S8), no genotype showed increased variance in more than one trait per G3 treatment, suggesting that ancestral salt stress overall had no major impact on phenotypic variance. Nevertheless, when we did find an effect, variance was usually increased in plants growing in a novel environment compared to the ancestral treatment, i.e. SSC in comparison to CCC; and CCS in comparison to SSS.

*Heat treatment* – In genotype Sha, heat treatment in G1 and G2 increased the number of rosette leaves at day 21 in G3, both in heat and control treatment (Table 2, Figure 5A), while none of the other genotypes showed a similar effect (Table 2). 

Two generations of ancestral heat treatment increased the variance of a number of traits for Cvi, both under G3 control and heat conditions when compared to plants with ancestral control treatment. Under both treatments these effects were moderate (p-values not significant after correction for multiple testing; Table 3; Figure 5C). None of the other genotypes showed increased variance for more than one trait (Table S9). Where increased variance was observed we tested whether G3 values correlated with values of their progenitors grown in G2. However, no such correlations could be observed, indicating that increased variance in G3 was independent of phenotypes in G2 (data not shown). 

**Table 3:** Effect of two generations of heat treatment (G1 and G2) vs two generations of control treatment on variances of traits measured under G3 heat and control conditions in genotype Cvi.

G3 treatment	Phenotypic trait	F_dF_	P		P^b^	
Heat	Rosette leaves d21	0.276_11,10_	0.046	*	0.114	
	Rosette leaves FFD	1.370_11,10_	0.627		0.697	
	Height	0.885_11,10_	0.839		0.839	
	Total siliques	0.252_11,10_	0.033	*	0.110	
	Mean silique length	0.217_11,10_	0.019	*	0.095	
Control	Rosette leaves d21	0.400_16,12_	0.089	·	0.178	
	Rosette leaves FFD	0.689_16,12_	0.479		0.599	
	Height	0.562_16,12_	0.280		0.400	
	Total siliques	0.232_16,12_	0.008	**	0.078	·
	Mean silique length	0.551_16,12_	0.264		0.400	

b P values were adjusted for multiple testing according to Benjamini and Hochberg [43].

** P value < 0.01; * P value < 0.05; ·:P value < 0.1

**Figure 6:**
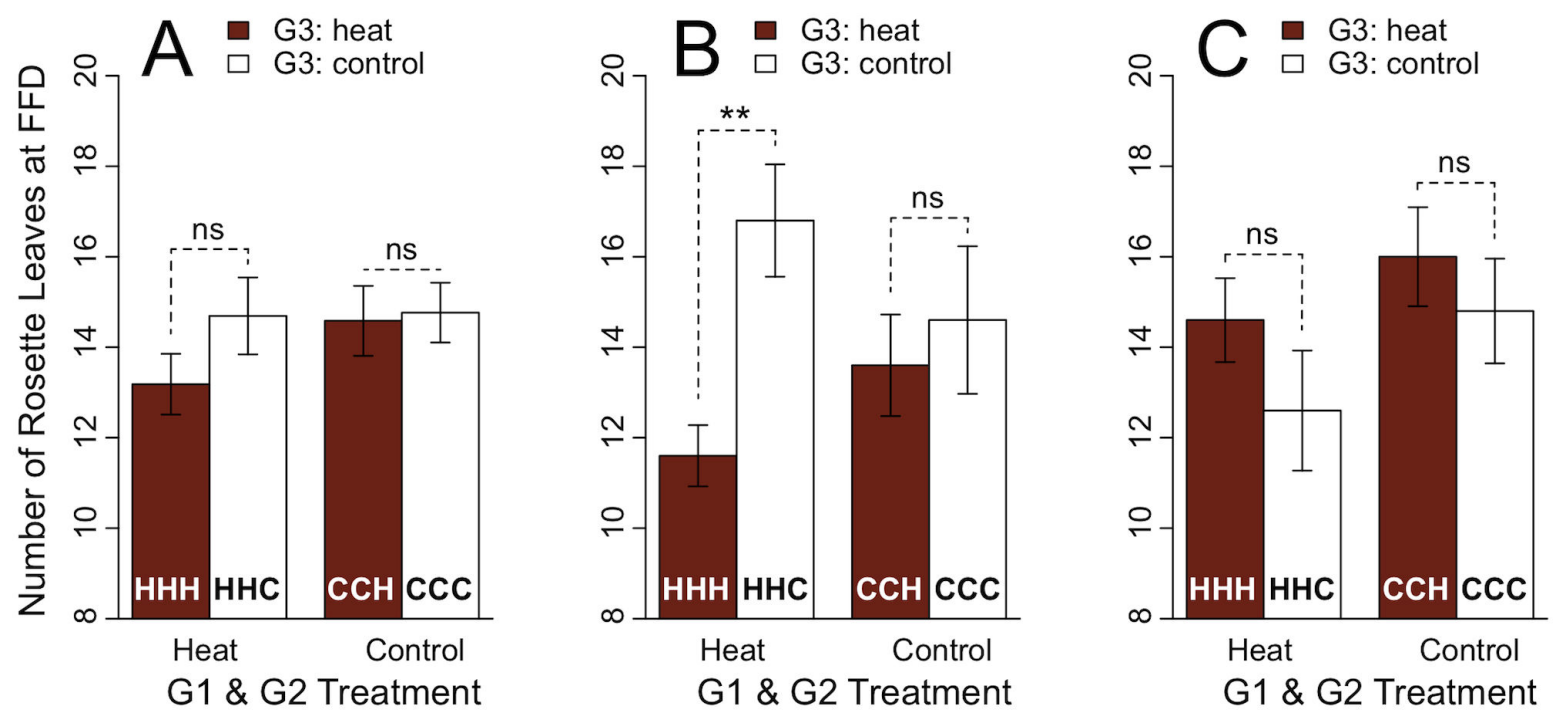
Transgenerational effects of heat on rosette leaves at FFD in genotype Cvi indicate diversified bet-hedging. A: Entire data-set. No differences in number of rosette leaves at FFD were found between G3 treatments when plants experienced the same ancestral treatment. B: For each G1G2 × G3-treatment combination the five plants with the highest silique number were chosen. In plants with ancestral heat treatment, number of rosette leaves differed significantly between G3 heat (HHH) and G3 control (HHC) treatment. When plants were control treated in G1 and G2, no differences were found between G3 treatments. C: For each G1G2 × G3-treatment combination the five plants with the lowest silique number were chosen. No differences in numbers of rosette leaves were found between G3 plants when plants experienced the same ancestral treatments.

We selected five Cvi plants with the highest fitness (number of siliques), each for HHH, HHC, CCH and CCC, to test whether increased variance due to ancestral heat stress (HHH and HHC) would allow different life history strategies to appear and be successful in different G3 environments. Of these five plants, those with ancestral heat treatment differed significantly in number of rosette leaves at FFD when grown in G3 heat treatment (HHH, few rosette leaves at FFD) compared to G3 control treatment (HHC, many rosette leaves at FFD; Figure 6B, left) indicating that through increased variance contrasting life history strategies could lead to highest fitness in different environments. No such effects were observed in plants with ancestral control treatment (Figure 6B, right), when the five plants with the lowest fitness were compared with each other (Figure 6C) or when the whole data set was used (Figure 6A). 

## Discussion

### Phenotypic responses to environmental stress within generations

*Salt* –Salt stress clearly reduced plant fitness, but genotypes differed substantially in the strength of this response. Genotypes Col and Ler responded strongly in most observed traits, whereas in genotype Sha fewer traits were responsive, and overall fitness seemed less affected, similar to previous studies [16,17]. Comparable to other studies [15,44] we also observed delayed flowering under saline conditions for Col and Ler. However, in Sha flowering was not delayed, making this genotype an interesting candidate to further study responses to saline conditions. 

*Heat* – Overall the phenotypic response to the applied heat treatment was weak, without decreasing overall fitness of heat treated plants. However, in three of the four genotypes, flowering was accelerated, similar to findings of Balasubramanian et al. [45], who grew about 100 *A. thaliana* accessions in slightly elevated temperatures (27 °C) and found induction of flowering due to elevated temperatures, although this effect varied extensively among accessions. Amongst the accession with moderate to strong induction were Col, Ler and Sha, corroborating our results. They further found that accessions with non-functional FRIGIDA (FRI) or FLOWERING LOCUS C (FLC) alleles showed induction of flowering, whereas accessions with functional *FLC* were insensitive to elevated temperatures. One exception was genotype Cvi, which has a non-functional *FRI* but nevertheless expresses *FLC* and as a result was insensitive to elevated temperatures. Because we also observed that Cvi was unresponsive to high temperatures, we assume that the same mechanism as described by Balasubramanian et al. [45] and Balasubramanian and Weigel [46] also induced flowering of Cvi in our experiments. However, there are important differences in our experimental setup compared to these earlier experiments. Balasubramanian et al. [45] grew their plants throughout the growth phase of the plant at only slightly elevated temperatures (27 °C) and short day conditions, whereas our plants grew mainly under control conditions (long day) and we applied heat stress only during three days at an early rosette stage by gradually increasing temperature to 40 °C to mimic hot days in natural environments. Such extreme temperatures are generally regarded as heat shock conditions for *A. thaliana*, although a gradual increase of temperature may affect plants differently than sudden exposure to high temperatures [6]. While it was proposed that heat shock may not induce flowering [45], we find that a relatively short exposure to high temperatures may suffice to effectively induce flowering in *A. thaliana*.

### Phenotypic effects of environmental stress across generations

Organisms can respond to changing environments in three different ways: a) tracking, i.e. a gradual change of mean trait values in response to altered natural selection in novel environments, b) phenotypic plasticity, i.e. different means of traits in different environments, or c) diversified bet-hedging, i.e. an increase of variance of a trait independent of the environment [33]. In our study we detected evidence for all three modes over the short time of three generations: ancestral heat conditions accelerated growth in genotype Sha, independent of G3 conditions, resembling a tracking response, while interaction between ancestral and G3 salt and control conditions led to increased number of siliques per branch in genotype Sha, comparable to transgenerational phenotypic plasticity. Furthermore, ancestral heat treatment increased the variance of a number of phenotypic traits in genotype Cvi, allowing for contrasting life history strategies to be successful under different G3 conditions, similar to diversified bet-hedging: while highest fitness under G3 heat conditions was reached by the fraction of offspring of heat treated plants that flowered earliest (i.e. with very few rosette leaves at FFD), the opposite fraction, i.e. the one with most rosette leaves at FFD, was fittest under G3 control conditions. Such different life history strategies were not found in offspring of control plants. However, it is important to emphasize that all these observations depended both on the type of stress applied as well as on the studied genotype, e.g. neither Ler nor Col exhibited any transgenerational phenotypic responses, suggesting that our findings of short-time phenotypic alterations may not be a general response providing adaptation to novel environments in *A. thaliana*.

Other studies addressing similar questions have found different traits to be responsive to transgenerational stress, however, we think that most of this can be assigned to different experimental designs. E.g. Whittle et al. [9] found higher fitness upon heat exposure due to ancestral heat stress, however, they used a design where plants were exposed to heat during seed development, which might affect plants very differently than the heat stress we applied during early vegetative growth. Similarly, Suter and Widmer [21] and Boyko et al. [47] found improved growth under salt conditions due to ancestral salt stress, suggesting an acquired tolerance to salt. Boyko et al. [47] used a very different approach by stressing plants in petri dishes, whereas Suter and Widmer [21] used more generations of stress, potentially reinforcing effects not observable after only three generations of stress.

By using a relatively large number of genetically virtually homozygous replications, we could exclude genetic diversification between stressed and control lines as important source of phenotypic variation. This suggests that a different mode of inheritance underlies the observed phenotypic changes in our study, potentially involving epigenetic processes. The finding of interaction between ancestral and G3 treatment could further corroborate the hypothesis of epigenetic inheritance: ancestral treatments could alter epigenetic patterns, which then are reversible upon exposure to present treatments (SSS differs from CCS, but SSC not from CCC in number of siliques per branch). Like in other studies suggesting similar epigenetic involvement in transgenerational phenotypic inheritance [9,21], further studies involving molecular analyses would be required to identify the molecular mechanisms of inheritance. 

Adaptation to environmental stress is often divided into tolerance and avoidance strategies. The former requires a plant to endure certain environmental conditions, whereas in the latter case a plant needs to sense adverse conditions and find a way to circumvent them. While the adaptive value of the transgenerational phenotypic changes observed in our climate chamber experiments is difficult to assess due to artificial conditions, we nevertheless propose that the observed effects of transgenerational phenotypic plasticity may be similar to avoidance strategies. SSS plants had more siliques per branch and grew taller than CCS plants, suggesting that the plants architecture underwent significant changes due to ancestral salt treatment. Similar phenotypic characteristics have been found to increase seed dispersal [48], which is one way how plants can affect their environment: if a plant grows under harsh conditions, dispersing seeds as far as possible may enhance chances of offspring to experience a more favorable environment [49]. Therefore, this altered architecture may help offspring of SSS plants to avoid saline conditions if the environment is sufficiently patchy and may therefore ultimately increase fitness.

The adaptive value of the tracking response is even more difficult to assess, as normally selection would be regarded as the driver of tracking [33], and no selection was consciously applied in this study. Nonetheless, we hypothesize that increased rosette growth due to ancestral heat treatment might indicate an acquired tolerance to heat stress, e.g. through stabilization of genes involved in the functioning of photosynthesis [50], known to be highly sensitive to heat stress [51]. This would allow the plant to grow faster even under elevated temperatures. Further studies taking e.g. the stability of Rubisco into account could clarify the underlying mechanisms.

The diversified bet-hedging resulting from ancestral heat treatment observed for genotype Cvi could be adaptive if different environmental conditions require contrasting phenotypes to reach maximal fitness, and if environments cannot be predicted reliably. This would ensure that at least a fraction of the offspring expresses the right phenotype in the encountered environment [33,36]. As the increased variance could not be explained by inheritance of extreme phenotypes in G2, we hypothesize that ancestral heat treatment may have destabilized epigenetic patterns. Increased epigenetic variation has recently been associated with a wider range of phenotypes [52], matching our observations. While bet-hedging is an evolutionary strategy often found under natural conditions in plants, e.g. in the timing of seed germination [37], *de novo* evolution of bet-hedging has, to our knowledge, so far mainly been studied in bacteria [36,53]. Further studies will have to establish under what conditions evolution or induction of bet-hedging can occur in plants and what molecular mechanisms are involved. 

In conclusion, we found that salt stress decreases fitness and delays flowering, while the exposure to heat early in the life cycle can accelerate flowering, although genotypes differ in the extent of their responses to stress. We also detected three different modes by which *A. thaliana* can respond to environmental change over the short time scale of three generations, although these were highly dependent on the type of stress as well as on the genotypes. We suggest that even in the absence of genetic variability, *A. thaliana* still maintains a surprisingly high potential to react to environmental stress and that these effects may at least partly be inherited to subsequent generations, potentially through epigenetic mechanisms. 

## Supporting Information

Figure S1 **Principal component analysis of the salt experiment, colored according to genotype.**
(PDF)

Table S1 **Setup of the pre-experiment to determine appropriate heat stress conditions.**
(DOCX)

Table S2 **Numbers of independent replicated lines that survived from G1 to G3 and the total number of plants that survived in G3.**
(DOCX)

Table S3 **Phenotypic traits measured in generation 3 (G3).**
(DOCX)

Table S4 **Mean ± standard deviation of phenotypic traits measured for four genotypes under heat and control conditions in G3.**
(DOCX)

Table S5 **Mean ± standard deviation of phenotypic traits measured for three genotypes under salt and control conditions in G3.**
(DOCX)

Table S6 **Effect of genotype, G3 heat treatment and G1G2 heat treatment and their pairwise interactions on phenotypic traits.**
(DOCX)

Table S7 **Effect of genotype, G3 salt treatment and G1G2 salt treatment and their pairwise interactions on phenotypic.**
(DOCX)

Table S8 **Effect of two generations of salt treatment (G1 and G2) vs. two generations of control treatment on variances of traits measured under G3 salt and control conditions.**
(DOCX)

Table S9 **Effect of two generations of heat treatment (G1 and G2) vs.** **two generations of control treatment on variances of traits measured under G3 heat and control conditions**.
(DOCX)
