# Supplementary figures and images for: Phenotypic Effects of Salt and Heat Stress over Three Generations in Arabidopsis thaliana
Source: PLoS One. 2013 Nov 14;8(11):e80819. doi: 10.1371/journal.pone.0080819 (PMC3828257; doi:10.1371/journal.pone.0080819)

**Figure S2:** Principal component analysis of the salt experiment coloured according to genotype.

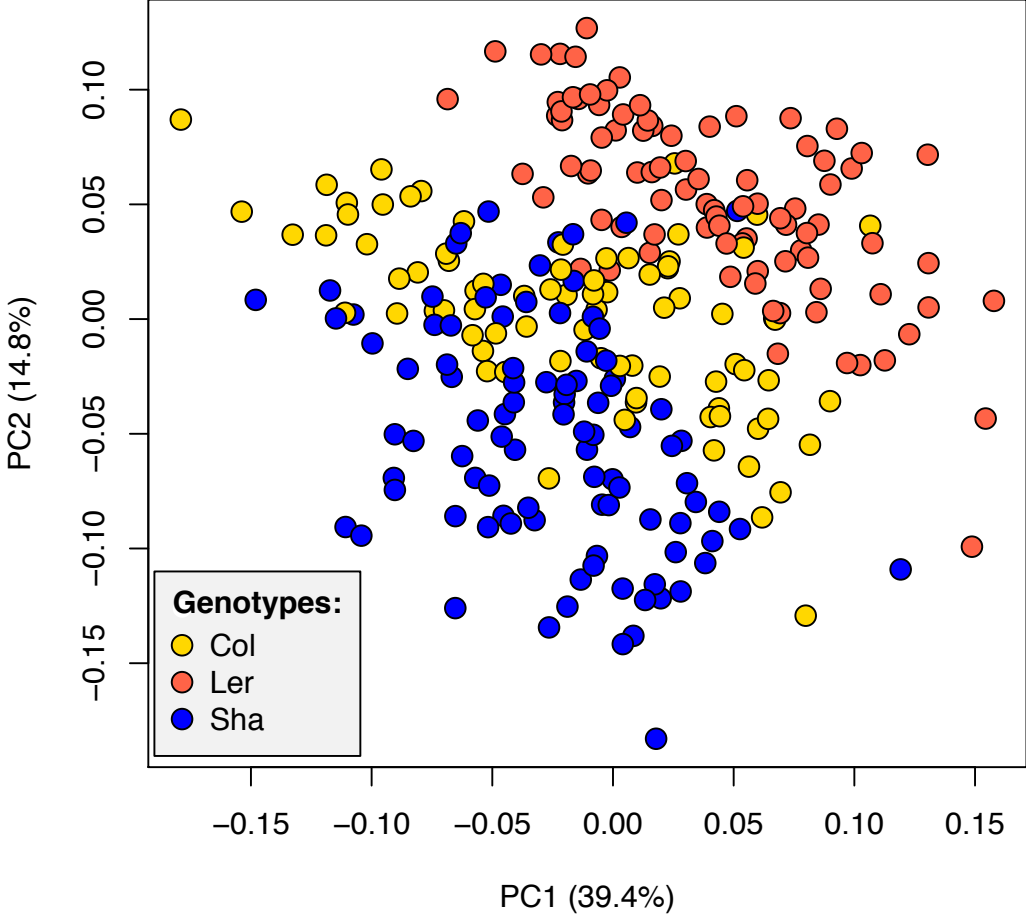

Supplement: Figure S1 — Principal component analysis of the salt experiment, colored according to genotype. (PDF) [file pone.0080819.s001.pdf]
