# Supplementary material for: Phenotypic Effects of Salt and Heat Stress over Three Generations in Arabidopsis thaliana
Source: PLoS One. 2013 Nov 14;8(11):e80819. doi: 10.1371/journal.pone.0080819 (PMC3828257; doi:10.1371/journal.pone.0080819)
Supplement: Table S1 — Setup of the pre-experiment to determine appropriate heat stress conditions. (DOCX) [file pone.0080819.s002.docx]

**Table S1**: Setup of the pre-experiment to determine appropriate heat stress conditions. Plants were grown under control conditions except on days when stress was applied. Night conditions were always identical to control conditions. When heat stress was applied, temperatures were gradually increased over 7 h from 18 °C to maximum temperature; this temperature was then held for 2 h and subsequently decreased gradually over 7 h to 18 °C. Light and humidity were identical to control conditions. Each of the 18 treatments was conducted using eight replica of genotype Col.

|  | Day 7 | Day 10 | Day 14 | Day 17 | Day 21 |
| --- | --- | --- | --- | --- | --- |
| 1 day stress, early | 32 °C | - | - | - | - |
|  | 36 °C | - | - | - | - |
|  | 40 °C | - | - | - | - |
| 1 day stress, late | - | - | 32 °C | - | - |
|  | - | - | 36 °C | - | - |
|  | - | - | 40 °C | - | - |
| 2 days stress, early | 32 °C | 32 °C | - | - | - |
|  | 36 °C | 36 °C | - | - | - |
|  | 40 °C | 40 °C | - | - | - |
| 2 days stress, late | - | - | 32 °C | 32 °C | - |
|  | - | - | 36 °C | 36 °C | - |
|  | - | - | 40 °C | 40 °C | - |
| 3 days stress, early | 32 °C | 32 °C | 32 °C | - | - |
|  | 36 °C | 36 °C | 36 °C | - | - |
|  | 40 °C | 40 °C | 40 °C | - | - |
| 3 days stress, late | - | - | 32 °C | 32 °C | 32 °C |
|  | - | - | 36 °C | 36 °C | 36 °C |
|  | - | - | 40 °C | 40 °C | 40 °C |
