# Supplementary material for: Phenotypic Effects of Salt and Heat Stress over Three Generations in Arabidopsis thaliana
Source: PLoS One. 2013 Nov 14;8(11):e80819. doi: 10.1371/journal.pone.0080819 (PMC3828257; doi:10.1371/journal.pone.0080819)
Supplement: Table S2 — Numbers of independent replicated lines that survived from G1 to G3 and the total number of plants that survived in G3. (DOCX) [file pone.0080819.s003.docx]

**Table S2:** Numbers of independent replicated lines that survived from G1 to G3 (“Lines”) and the total number of plants that survived in G3 (“Plants”), listed separately for each genotype and treatment. Initially, 25 replica were grown per treatment. “% Lines” indicates the average percentage of independent lines per genotype, separately for the heat and salt experiments.

|  |  | **Heat experiment** | | | | | | | | |  |
| --- | --- | --- | --- | --- | --- | --- | --- | --- | --- | --- | --- |
|  |  | **HHH** | **CHH** | **HCH** | **CCH** | **HHC** | **CHC** | **HCC** | **CCC** | **% Lines** | |
| **Col** | Lines | 16 | 14 | 15 | 18 | 16 | 14 | 13 | 16 | 77.7 | |
|  | **Plants** | **19** | **20** | **20** | **21** | **19** | **20** | **20** | **18** |  | |
| **Cvi** | Lines | 7 | 5 | 6 | 8 | 10 | 8 | 9 | 11 | 66.7 | |
|  | **Plants** | **11** | **8** | **9** | **12** | **13** | **14** | **12** | **17** |  | |
| **Ler** | Lines | 11 | 19 | 12 | 12 | 8 | 11 | 12 | 12 | 70.8 | |
|  | **Plants** | **18** | **22** | **19** | **15** | **19** | **11** | **18** | **15** |  | |
| **Sha** | Lines | 21 | 21 | 19 | 16 | 22 | 20 | 18 | 17 | 81.1 | |
|  | **Plants** | **23** | **24** | **25** | **22** | **25** | **24** | **23** | **24** |  | |
|  |  |  |  |  |  |  |  |  |  |  | |
|  |  |  |  |  |  |  |  |  |  |  | |
|  |  | **Salt experiment** | | | | | | | | |  |
|  |  | **SSS** | **CSS** | **SCS** | **CCS** | **SSC** | **CSC** | **SCC** | **CCC** | **% Lines** | |
| **Col** | Lines | 12 | 9 | 14 | 20 | 15 | 10 | 16 | 21 | 68.0 | |
|  | **Plants** | **16** | **17** | **20** | **21** | **25** | **25** | **25** | **23** |  | |
| **Ler** | Lines | 9 | 5 | 9 | 13 | 9 | 5 | 9 | 14 | 42.0 | |
|  | **Plants** | **22** | **16** | **14** | **22** | **25** | **25** | **25** | **25** |  | |
| **Sha** | Lines | 15 | 10 | 17 | 20 | 15 | 10 | 17 | 20 | 64.9 | |
|  | **Plants** | **23** | **23** | **23** | **24** | **24** | **25** | **24** | **25** |  | |
