# Supplementary material for: Phenotypic Effects of Salt and Heat Stress over Three Generations in Arabidopsis thaliana
Source: PLoS One. 2013 Nov 14;8(11):e80819. doi: 10.1371/journal.pone.0080819 (PMC3828257; doi:10.1371/journal.pone.0080819)
Supplement: Table S3 — Phenotypic traits measured in generation 3 (G3). (DOCX) [file pone.0080819.s004.docx]

**Table S3**: Phenotypic traits measured in generation 3 (G3).

| **Phenotypic trait** | **Time measured** | **Calculated as** |
| --- | --- | --- |
| Rosette diameter | Day 14, day 21 and FFD | - |
| Number of rosette leaves | Day 14, day 21 and FFD | - |
| Flowering | FFD | - |
| Number of cauline leaves | FFD | - |
| Final height | Day 70 | - |
| Lenth of main stem (first to last silique) | After harvest |  |
| Siliques at main stem | After harvest |  |
| Total siliques | After harvest | - |
| Side-branches main stem | After harvest |  |
| Total branches | After harvest |  |
| Siliques per side-branch | After harvest, computed | (Total siliques - siliques at main stem) / (total branches - 1) |
| Distance between siliques | After harvest, computed | Distance between lowest and highest siliques of main stem / (siliques at mainstem - 1) |
| Mean silique length | After harvest, computed | Average of lengths of 3^rd^, 5^th^ and 7^th^ silique from below at main stem |
| Total silique length | After harvest, computed | Total siliques ✕ average length of siliques |
