# Supplementary material for: Phenotypic Effects of Salt and Heat Stress over Three Generations in Arabidopsis thaliana
Source: PLoS One. 2013 Nov 14;8(11):e80819. doi: 10.1371/journal.pone.0080819 (PMC3828257; doi:10.1371/journal.pone.0080819)
Supplement: Table S4 — Mean ± standard deviation of phenotypic traits measured for four genotypes under heat and control conditions in G3. (DOCX) [file pone.0080819.s005.docx]

Table S4: Mean ± standard deviation of phenotypic traits measured for four genotypes (gen.) under heat and control conditions in generation 3 (G3). HHH: G1, G2 and G3 heat treated; CCH: G1 and G2 control treated, G3 heat treated; HHC: G1 and G2 heat treated, G3 control treated, CCC: G1, G2 and G3 control treated.

| **Gen.** | **Phenotypic trait** | **HHH** | | | **CCH** | | | **HHC** | | | **CCC** | | |
| --- | --- | --- | --- | --- | --- | --- | --- | --- | --- | --- | --- | --- | --- |
| Col | Rosette diameter 2 weeks [mm] | 14.526 | ± | 2.988 | 14.429 | ± | 3.124 | 15.211 | ± | 3.675 | 16.833 | ± | 4.076 |
|  | Rosette leaves 2 weeks | 4.526 | ± | 0.697 | 4.762 | ± | 0.700 | 4.842 | ± | 0.958 | 5.167 | ± | 0.857 |
|  | Rosette diameter 3 weeks [mm] | 38.263 | ± | 7.125 | 40.381 | ± | 7.768 | 40.842 | ± | 9.587 | 43.444 | ± | 9.636 |
|  | Rosette leaves 3 weeks | 11.368 | ± | 1.165 | 11.714 | ± | 1.384 | 11.947 | ± | 1.471 | 12.556 | ± | 1.790 |
|  | FFD | 31.263 | ± | 1.851 | 30.714 | ± | 2.028 | 32.421 | ± | 2.219 | 31.556 | ± | 2.595 |
|  | Rosette diameter FFD [mm] | 107.579 | ± | 9.822 | 107.857 | ± | 10.331 | 116.947 | ± | 11.183 | 116.167 | ± | 11.521 |
|  | Rosette leaves FFD | 15.105 | ± | 1.524 | 15.286 | ± | 1.586 | 17.526 | ± | 2.270 | 17.722 | ± | 1.776 |
|  | Cauline leaves FFD | 3.158 | ± | 0.958 | 2.952 | ± | 0.740 | 4.368 | ± | 0.684 | 4.222 | ± | 0.878 |
|  | Height [mm] | 512.263 | ± | 64.492 | 523.667 | ± | 45.044 | 508.842 | ± | 42.008 | 519.278 | ± | 58.492 |
|  | Length main stem [mm] | 446.158 | ± | 84.714 | 423.238 | ± | 88.549 | 389.579 | ± | 72.232 | 402.889 | ± | 65.359 |
|  | Siliques at main stem | 61.158 | ± | 17.189 | 57.048 | ± | 12.929 | 59.737 | ± | 17.562 | 65.167 | ± | 17.757 |
|  | Distance between siliques [mm] | 7.828 | ± | 2.252 | 7.639 | ± | 0.955 | 7.028 | ± | 1.843 | 6.611 | ± | 1.628 |
|  | Side-branches main stem | 6.842 | ± | 1.772 | 7.190 | ± | 1.123 | 6.895 | ± | 1.410 | 7.167 | ± | 1.425 |
|  | Total branches | 42.895 | ± | 13.212 | 41.190 | ± | 14.607 | 46.105 | ± | 11.460 | 44.611 | ± | 16.660 |
|  | Siliques per branch | 16.779 | ± | 2.917 | 18.863 | ± | 4.255 | 15.732 | ± | 3.625 | 17.478 | ± | 8.286 |
|  | Total siliques | 737.526 | ± | 151.537 | 772.333 | ± | 183.306 | 753.368 | ± | 189.636 | 719.333 | ± | 152.485 |
|  | Mean length siliques [mm] | 17.632 | ± | 1.071 | 17.603 | ± | 0.970 | 17.140 | ± | 0.723 | 17.278 | ± | 1.373 |
|  | Total silique length [cm] | 1296.912 | ± | 259.705 | 1353.148 | ± | 307.077 | 1292.719 | ± | 334.243 | 1249.700 | ± | 313.505 |
| Ler | Rosette diameter 2 weeks [mm] | 10.500 | ± | 2.618 | 10.714 | ± | 1.939 | 10.526 | ± | 3.963 | 10.429 | ± | 1.828 |
|  | Rosette leaves 2 weeks | 3.611 | ± | 0.916 | 3.786 | ± | 0.579 | 3.211 | ± | 1.512 | 3.429 | ± | 1.158 |
|  | Rosette diameter 3 weeks [mm] | 33.389 | ± | 10.461 | 33.714 | ± | 6.069 | 31.789 | ± | 7.323 | 32.786 | ± | 5.846 |
|  | Rosette leaves 3 weeks | 8.889 | ± | 1.605 | 8.929 | ± | 0.730 | 8.737 | ± | 1.851 | 9.286 | ± | 0.825 |
|  | FFD | 30.167 | ± | 2.684 | 29.714 | ± | 1.729 | 30.737 | ± | 1.821 | 30.714 | ± | 1.204 |
|  | Rosette diameter FFD [mm] | 79.056 | ± | 7.996 | 79.786 | ± | 10.460 | 84.632 | ± | 6.020 | 86.500 | ± | 8.225 |
|  | Rosette leaves FFD | 9.500 | ± | 0.985 | 9.571 | ± | 0.646 | 10.316 | ± | 0.946 | 10.571 | ± | 1.016 |
|  | Cauline leaves FFD | 3.000 | ± | 0.594 | 2.786 | ± | 0.579 | 3.526 | ± | 0.612 | 3.500 | ± | 0.760 |
|  | Height [mm] | 375.833 | ± | 31.448 | 387.643 | ± | 22.359 | 374.842 | ± | 31.136 | 374.857 | ± | 25.681 |
|  | Length main stem [mm] | 318.722 | ± | 79.856 | 314.286 | ± | 60.857 | 278.053 | ± | 68.658 | 297.357 | ± | 41.135 |
|  | Siliques at main stem | 52.778 | ± | 19.672 | 52.714 | ± | 7.946 | 59.000 | ± | 16.371 | 59.643 | ± | 20.556 |
|  | Distance between siliques [mm] | 6.628 | ± | 2.134 | 6.151 | ± | 1.352 | 5.103 | ± | 1.611 | 5.711 | ± | 2.302 |
|  | Side-branches main stem | 7.111 | ± | 1.183 | 7.143 | ± | 0.864 | 7.579 | ± | 1.677 | 7.214 | ± | 1.311 |
|  | Total branches | 35.278 | ± | 13.594 | 30.071 | ± | 5.015 | 34.263 | ± | 11.874 | 33.071 | ± | 12.307 |
|  | Siliques per branch | 16.808 | ± | 3.661 | 16.575 | ± | 2.698 | 17.241 | ± | 4.998 | 16.718 | ± | 4.107 |
|  | Total siliques | 592.111 | ± | 126.698 | 527.786 | ± | 77.896 | 593.211 | ± | 118.696 | 562.857 | ± | 119.986 |
|  | Mean length siliques [mm] | 14.537 | ± | 1.042 | 14.119 | ± | 1.129 | 14.351 | ± | 0.789 | 14.762 | ± | 0.745 |
|  | Total silique length [cm] | 857.361 | ± | 178.702 | 747.071 | ± | 136.959 | 854.277 | ± | 189.897 | 834.152 | ± | 198.829 |
| Cvi | Rosette diameter 2 weeks [mm] | 13.818 | ± | 4.423 | 14.500 | ± | 1.834 | 10.615 | ± | 3.330 | 12.176 | ± | 2.856 |
|  | Rosette leaves 2 weeks | 3.455 | ± | 0.820 | 3.833 | ± | 0.389 | 3.077 | ± | 1.256 | 3.235 | ± | 0.752 |
|  | Rosette diameter 3 weeks [mm] | 32.545 | ± | 8.383 | 34.500 | ± | 3.090 | 27.308 | ± | 9.205 | 31.176 | ± | 6.317 |
|  | Rosette leaves 3 weeks | 7.818 | ± | 1.401 | 8.083 | ± | 0.669 | 6.462 | ± | 1.561 | 7.059 | ± | 1.029 |
|  | FFD | 35.091 | ± | 3.646 | 36.083 | ± | 2.843 | 38.538 | ± | 4.926 | 36.941 | ± | 2.680 |
|  | Rosette diameter FFD [mm] | 105.273 | ± | 12.076 | 110.917 | ± | 8.005 | 103.000 | ± | 13.329 | 107.941 | ± | 8.613 |
|  | Rosette leaves FFD | 13.182 | ± | 2.228 | 14.583 | ± | 2.678 | 14.692 | ± | 3.066 | 14.765 | ± | 2.728 |
|  | Cauline leaves FFD | 3.000 | ± | 0.775 | 3.500 | ± | 1.000 | 3.692 | ± | 0.855 | 3.647 | ± | 0.786 |
|  | Height [mm] | 425.000 | ± | 55.474 | 418.000 | ± | 59.356 | 392.846 | ± | 63.808 | 386.941 | ± | 49.736 |
|  | Length main stem [mm] | 311.364 | ± | 91.658 | 327.167 | ± | 116.520 | 249.154 | ± | 69.952 | 228.824 | ± | 62.561 |
|  | Siliques at main stem | 44.727 | ± | 10.238 | 41.583 | ± | 8.163 | 39.923 | ± | 10.820 | 36.294 | ± | 10.475 |
|  | Distance between siliques [mm] | 7.290 | ± | 2.138 | 8.108 | ± | 2.369 | 6.568 | ± | 1.637 | 6.702 | ± | 1.567 |
|  | Side-branches main stem | 7.545 | ± | 1.128 | 8.667 | ± | 2.270 | 7.769 | ± | 2.488 | 7.294 | ± | 2.144 |
|  | Total branches | 29.727 | ± | 10.799 | 31.417 | ± | 9.219 | 25.769 | ± | 11.770 | 32.765 | ± | 9.757 |
|  | Siliques per branch | 14.366 | ± | 4.998 | 11.500 | ± | 3.517 | 13.221 | ± | 3.201 | 12.960 | ± | 4.848 |
|  | Total siliques | 430.818 | ± | 140.695 | 367.083 | ± | 65.572 | 357.769 | ± | 145.355 | 409.529 | ± | 68.978 |
|  | Mean length siliques [mm] | 21.515 | ± | 2.460 | 22.000 | ± | 1.110 | 22.256 | ± | 1.891 | 21.824 | ± | 1.434 |
|  | Total silique length [cm] | 906.403 | ± | 212.661 | 808.622 | ± | 153.668 | 811.969 | ± | 380.717 | 894.316 | ± | 166.617 |
| Sha | Rosette diameter 2 weeks [mm] | 17.000 | ± | 2.449 | 14.818 | ± | 4.500 | 17.520 | ± | 2.931 | 15.333 | ± | 4.351 |
|  | Rosette leaves 2 weeks | 4.261 | ± | 0.449 | 4.045 | ± | 0.575 | 4.840 | ± | 0.473 | 4.583 | ± | 0.584 |
|  | Rosette diameter 3 weeks [mm] | 44.261 | ± | 5.038 | 41.545 | ± | 8.210 | 47.040 | ± | 5.660 | 41.542 | ± | 8.193 |
|  | Rosette leaves 3 weeks | 10.043 | ± | 0.928 | 9.273 | ± | 1.202 | 10.640 | ± | 0.810 | 9.917 | ± | 1.176 |
|  | FFD | 29.174 | ± | 0.984 | 29.864 | ± | 2.253 | 30.560 | ± | 1.850 | 31.167 | ± | 1.926 |
|  | Rosette diameter FFD [mm] | 94.696 | ± | 7.882 | 88.318 | ± | 19.115 | 105.400 | ± | 10.231 | 102.208 | ± | 7.442 |
|  | Rosette leaves FFD | 10.609 | ± | 1.234 | 10.545 | ± | 1.819 | 13.120 | ± | 1.965 | 12.208 | ± | 1.382 |
|  | Cauline leaves FFD | 2.522 | ± | 0.511 | 2.409 | ± | 0.734 | 3.000 | ± | 0.577 | 3.125 | ± | 0.612 |
|  | Height [mm] | 560.913 | ± | 98.481 | 580.864 | ± | 102.737 | 532.360 | ± | 66.723 | 571.083 | ± | 68.348 |
|  | Length main stem [mm] | 486.565 | ± | 120.371 | 517.682 | ± | 122.477 | 456.960 | ± | 96.802 | 516.000 | ± | 103.378 |
|  | Siliques at main stem | 69.391 | ± | 17.175 | 71.773 | ± | 19.138 | 68.880 | ± | 15.528 | 75.667 | ± | 14.264 |
|  | Distance between siliques [mm] | 7.260 | ± | 1.255 | 7.546 | ± | 1.560 | 6.944 | ± | 1.803 | 6.976 | ± | 1.071 |
|  | Side-branches main stem | 7.174 | ± | 1.586 | 7.227 | ± | 1.193 | 7.920 | ± | 1.470 | 7.542 | ± | 1.414 |
|  | Total branches | 44.261 | ± | 11.913 | 42.045 | ± | 15.051 | 40.720 | ± | 12.432 | 37.958 | ± | 10.869 |
|  | Siliques per branch | 20.751 | ± | 4.286 | 22.141 | ± | 3.876 | 21.527 | ± | 3.876 | 22.593 | ± | 4.402 |
|  | Total siliques | 934.391 | ± | 190.237 | 935.455 | ± | 211.256 | 894.480 | ± | 184.457 | 879.500 | ± | 177.238 |
|  | Mean length siliques [mm] | 14.464 | ± | 0.983 | 14.591 | ± | 1.182 | 14.333 | ± | 0.694 | 14.500 | ± | 0.958 |
|  | Total silique length [cm] | 1351.803 | ± | 296.469 | 1367.500 | ± | 330.413 | 1280.184 | ± | 261.106 | 1270.350 | ± | 242.163 |
