# Supplementary material for: Phenotypic Effects of Salt and Heat Stress over Three Generations in Arabidopsis thaliana
Source: PLoS One. 2013 Nov 14;8(11):e80819. doi: 10.1371/journal.pone.0080819 (PMC3828257; doi:10.1371/journal.pone.0080819)
Supplement: Table S5 — Mean ± standard deviation of phenotypic traits measured for three genotypes under salt and control conditions in G3. (DOCX) [file pone.0080819.s006.docx]

Table S5: Mean ± standard deviation of phenotypic traits measured for three genotypes (gen.) under salt and control conditions in generation 3 (G3). SSS: G1, G2 and G3 salt treated; CCS: G1 and G2 control treated, G3 salt treated; SSC: G1 and G2 salt treated, G3 control treated, CCC: G1, G2 and G3 control treated.

| **Gen.** | **Phenotypic trait** | **SSS** | | | **CCS** | | | **SSC** | | | **CCC** | | |
| --- | --- | --- | --- | --- | --- | --- | --- | --- | --- | --- | --- | --- | --- |
| Col | Rosette diameter 2 weeks [mm] | 11.812 | ± | 4.665 | 12.500 | ± | 3.269 | 15.960 | ± | 5.488 | 15.217 | ± | 5.317 |
|  | Rosette leaves 2 weeks | 4.375 | ± | 1.088 | 4.500 | ± | 1.000 | 4.960 | ± | 1.369 | 5.043 | ± | 1.331 |
|  | Rosette diameter 3 weeks [mm] | 21.688 | ± | 7.812 | 24.700 | ± | 5.602 | 34.720 | ± | 12.908 | 32.000 | ± | 13.919 |
|  | Rosette leaves 3 weeks | 9.250 | ± | 1.915 | 9.900 | ± | 1.294 | 11.120 | ± | 2.223 | 10.913 | ± | 2.372 |
|  | FFD | 36.875 | ± | 6.302 | 35.750 | ± | 3.041 | 31.880 | ± | 4.503 | 31.087 | ± | 3.204 |
|  | Rosette diameter FFD [mm] | 64.062 | ± | 10.969 | 63.150 | ± | 8.028 | 69.640 | ± | 19.697 | 66.043 | ± | 21.244 |
|  | Rosette leaves FFD | 18.562 | ± | 3.183 | 17.850 | ± | 2.519 | 15.680 | ± | 2.193 | 14.391 | ± | 2.607 |
|  | Cauline leaves FFD | 4.250 | ± | 1.000 | 4.200 | ± | 0.768 | 3.800 | ± | 0.707 | 3.826 | ± | 1.072 |
|  | Height [mm] | 345.312 | ± | 55.740 | 346.750 | ± | 67.001 | 492.120 | ± | 48.119 | 470.565 | ± | 48.778 |
|  | Length main stem [mm] | 261.875 | ± | 48.246 | 264.700 | ± | 59.707 | 373.480 | ± | 52.306 | 362.043 | ± | 54.969 |
|  | Siliques at main stem | 46.750 | ± | 5.825 | 45.200 | ± | 8.076 | 53.040 | ± | 6.895 | 51.130 | ± | 9.167 |
|  | Distance between siliques [mm] | 5.703 | ± | 0.680 | 5.979 | ± | 0.775 | 7.264 | ± | 1.125 | 7.369 | ± | 1.343 |
|  | Side-branches main stem | 2.562 | ± | 1.094 | 2.350 | ± | 0.813 | 4.440 | ± | 1.873 | 4.087 | ± | 1.379 |
|  | Total branches | 11.625 | ± | 5.737 | 12.200 | ± | 5.736 | 27.120 | ± | 15.904 | 23.087 | ± | 7.166 |
|  | Siliques per branch | 15.457 | ± | 4.440 | 14.693 | ± | 4.530 | 17.269 | ± | 4.494 | 15.966 | ± | 3.809 |
|  | Total siliques | 197.000 | ± | 74.581 | 203.500 | ± | 84.409 | 476.080 | ± | 217.997 | 403.130 | ± | 136.430 |
|  | Mean length siliques [mm] | 14.562 | ± | 1.348 | 14.083 | ± | 2.714 | 15.893 | ± | 0.891 | 15.812 | ± | 0.846 |
|  | Total silique length [cm] | 287.471 | ± | 118.796 | 293.445 | ± | 140.703 | 770.272 | ± | 394.872 | 644.616 | ± | 241.739 |
| Ler | Rosette diameter 2 weeks [mm] | 9.524 | ± | 2.337 | 9.300 | ± | 2.473 | 10.720 | ± | 3.932 | 10.583 | ± | 2.448 |
|  | Rosette leaves 2 weeks | 3.810 | ± | 0.981 | 3.750 | ± | 0.786 | 3.720 | ± | 1.568 | 3.917 | ± | 0.717 |
|  | Rosette diameter 3 weeks [mm] | 20.429 | ± | 6.063 | 19.750 | ± | 6.874 | 25.600 | ± | 11.273 | 27.042 | ± | 8.755 |
|  | Rosette leaves 3 weeks | 8.143 | ± | 1.195 | 7.800 | ± | 1.322 | 8.040 | ± | 2.574 | 8.333 | ± | 1.834 |
|  | FFD | 34.333 | ± | 4.768 | 35.250 | ± | 4.678 | 31.280 | ± | 5.374 | 30.417 | ± | 3.387 |
|  | Rosette diameter FFD [mm] | 49.714 | ± | 10.233 | 50.850 | ± | 9.190 | 53.920 | ± | 14.445 | 59.208 | ± | 16.590 |
|  | Rosette leaves FFD | 10.952 | ± | 1.161 | 11.750 | ± | 2.149 | 9.360 | ± | 1.254 | 9.250 | ± | 1.189 |
|  | Cauline leaves FFD | 3.571 | ± | 0.870 | 3.700 | ± | 0.865 | 2.960 | ± | 0.611 | 3.250 | ± | 0.676 |
|  | Height [mm] | 252.429 | ± | 53.648 | 245.050 | ± | 44.106 | 313.160 | ± | 32.189 | 322.500 | ± | 35.620 |
|  | Length main stem [mm] | 166.048 | ± | 45.913 | 151.800 | ± | 35.283 | 208.400 | ± | 38.167 | 216.958 | ± | 46.376 |
|  | Siliques at main stem | 44.571 | ± | 12.624 | 44.550 | ± | 12.011 | 49.320 | ± | 6.473 | 49.750 | ± | 6.367 |
|  | Distance between siliques [mm] | 4.088 | ± | 1.333 | 3.789 | ± | 1.735 | 4.334 | ± | 0.672 | 4.452 | ± | 0.800 |
|  | Side-branches main stem | 2.429 | ± | 1.568 | 2.350 | ± | 1.089 | 4.560 | ± | 2.043 | 5.083 | ± | 1.840 |
|  | Total branches | 7.286 | ± | 3.052 | 6.350 | ± | 2.390 | 22.520 | ± | 13.355 | 21.750 | ± | 11.395 |
|  | Siliques per branch | 22.624 | ± | 18.199 | 19.276 | ± | 7.664 | 16.207 | ± | 6.477 | 16.618 | ± | 5.918 |
|  | Total siliques | 179.429 | ± | 93.914 | 148.700 | ± | 68.089 | 343.440 | ± | 178.409 | 353.917 | ± | 127.583 |
|  | Mean length siliques [mm] | 13.397 | ± | 2.575 | 12.900 | ± | 1.774 | 13.467 | ± | 0.833 | 13.764 | ± | 0.955 |
|  | Total silique length [cm] | 227.949 | ± | 99.096 | 197.330 | ± | 99.135 | 472.160 | ± | 272.029 | 489.637 | ± | 183.864 |
| Sha | Rosette diameter 2 weeks [mm] | 14.565 | ± | 3.740 | 15.750 | ± | 4.214 | 16.542 | ± | 4.587 | 15.720 | ± | 4.523 |
|  | Rosette leaves 2 weeks | 4.565 | ± | 0.662 | 4.458 | ± | 0.509 | 4.500 | ± | 0.885 | 4.280 | ± | 0.843 |
|  | Rosette diameter 3 weeks [mm] | 31.478 | ± | 8.174 | 31.500 | ± | 6.567 | 36.625 | ± | 10.274 | 35.080 | ± | 10.161 |
|  | Rosette leaves 3 weeks | 8.783 | ± | 0.998 | 9.333 | ± | 0.963 | 8.792 | ± | 1.444 | 9.120 | ± | 1.536 |
|  | FFD | 32.739 | ± | 3.333 | 33.792 | ± | 3.811 | 32.125 | ± | 6.271 | 31.680 | ± | 2.911 |
|  | Rosette diameter FFD [mm] | 63.478 | ± | 12.537 | 63.875 | ± | 8.724 | 67.583 | ± | 13.038 | 71.200 | ± | 19.617 |
|  | Rosette leaves FFD | 12.739 | ± | 2.200 | 14.000 | ± | 3.362 | 12.583 | ± | 3.611 | 12.000 | ± | 2.432 |
|  | Cauline leaves FFD | 3.130 | ± | 0.815 | 3.833 | ± | 1.049 | 3.083 | ± | 0.929 | 2.920 | ± | 0.812 |
|  | Height [mm] | 482.957 | ± | 70.131 | 443.500 | ± | 61.035 | 604.500 | ± | 79.831 | 584.360 | ± | 75.349 |
|  | Length main stem [mm] | 410.087 | ± | 72.592 | 358.500 | ± | 84.548 | 510.333 | ± | 103.407 | 505.760 | ± | 103.745 |
|  | Siliques at main stem | 68.826 | ± | 12.067 | 62.167 | ± | 15.728 | 73.833 | ± | 13.849 | 71.360 | ± | 15.607 |
|  | Distance between siliques [mm] | 6.087 | ± | 0.716 | 5.961 | ± | 0.899 | 7.089 | ± | 1.130 | 7.347 | ± | 1.388 |
|  | Side-branches main stem | 3.261 | ± | 1.888 | 2.750 | ± | 1.422 | 4.208 | ± | 2.167 | 4.920 | ± | 1.824 |
|  | Total branches | 9.304 | ± | 4.724 | 9.833 | ± | 3.773 | 18.333 | ± | 9.832 | 17.920 | ± | 8.583 |
|  | Siliques per branch | 29.015 | ± | 8.853 | 24.260 | ± | 8.012 | 23.204 | ± | 5.511 | 24.248 | ± | 5.198 |
|  | Total siliques | 279.522 | ± | 67.647 | 256.042 | ± | 60.989 | 463.000 | ± | 223.571 | 467.280 | ± | 158.507 |
|  | Mean length siliques [mm] | 14.159 | ± | 0.989 | 14.194 | ± | 0.735 | 13.889 | ± | 0.562 | 13.973 | ± | 0.827 |
|  | Total silique length [mm] | 399.172 | ± | 113.055 | 365.707 | ± | 98.572 | 647.118 | ± | 325.141 | 660.807 | ± | 248.960 |
