# Supplementary material for: Phenotypic Effects of Salt and Heat Stress over Three Generations in Arabidopsis thaliana
Source: PLoS One. 2013 Nov 14;8(11):e80819. doi: 10.1371/journal.pone.0080819 (PMC3828257; doi:10.1371/journal.pone.0080819)
Supplement: Table S6 — Effect of genotype, G3 heat treatment and G1G2 heat treatment and their pairwise interactions on phenotypic traits. (DOCX) [file pone.0080819.s007.docx]

Table S4: Effect of Genotype, G3 heat treatment and G1G2 heat treatment and their pairwise interactions on phenotypic traits calculated using a linear mixed model with tray as random factor.

|  |  | Genotype | |  | G3 treatment | |  | G1G2 treatment | |  | Genotype x G3 | |  | Genotype x G1G2 | |  | G3 x G1G2 | |
| --- | --- | --- | --- | --- | --- | --- | --- | --- | --- | --- | --- | --- | --- | --- | --- | --- | --- | --- |
| Phenotypic traits |  | F_dF_ | P^b^ |  | F_dF_ | P^b^ |  | F_dF_ | P^b^ |  | F_dF_ | P^b^ |  | F_dF_ | P^b^ |  | F_dF_ | P^b^ |
| Rosette leaves 3 weeks |  | **48.336_3,248_** | **<0.001** |  | 0.279_1,28_ | 0.702 |  | 0.126_1,248_ | 0.723 |  | 1.402_3,248_ | 0.425 |  | 3.973_3,248_ | 0.020 |  | 0.506_1,248_ | 0.668 |
| Rosette leaves FFD |  | **92.633_3,248_** | **<0.001** |  | **26.845_1,28_** | **<0.001** |  | 0.160_1,248_ | 0.689 |  | 2.208_3,248_ | 0.154 |  | 1.612_3,248_ | 0.262 |  | 1.257_1,248_ | 0.307 |
| Height |  | **45.005_3,248_** | **<0.001** |  | 0.879_1,28_ | 0.545 |  | 1.879_1,248_ | 0.401 |  | 0.851_3,248_ | 0.545 |  | 0.513_3,248_ | 0.674 |  | 0.710_1,248_ | 0.545 |
| Total siliques |  | **53.477_3,248_** | **<0.001** |  | 0.174_1,28_ | 0.951 |  | 0.018_1,248_ | 0.980 |  | 0.062_3,248_ | 0.980 |  | 0.727_3,248_ | 0.951 |  | 0.261_1,248_ | 0.951 |
| Mean length siliques |  | **303.523_3,248_** | **<0.001** |  | 0.028_1,28_ | 0.986 |  | 0.108_1,248_ | 0.986 |  | 0.714_3,248_ | 0.981 |  | 0.048_3,248_ | 0.986 |  | 0.340_1,248_ | 0.981 |

^b^P-values were adjusted for multiple testing according to Benjamini and Hochberg (1995)
