# Supplementary material for: Phenotypic Effects of Salt and Heat Stress over Three Generations in Arabidopsis thaliana
Source: PLoS One. 2013 Nov 14;8(11):e80819. doi: 10.1371/journal.pone.0080819 (PMC3828257; doi:10.1371/journal.pone.0080819)
Supplement: Table S7 — Effect of genotype, G3 salt treatment and G1G2 salt treatment and their pairwise interactions on phenotypic. (DOCX) [file pone.0080819.s008.docx]

Table S5: Effect of Genotype, G3 salt treatment and G1G2 salt treatment and their pairwise interactions on phenotypic traits calculated using a linear mixed model with tray as random factor.

|  |  | Genotype | |  | G3 treatment | |  | G1G2 treatment | |  | Genotype x G3 | |  | Genotype x G1G2 | |  | G3 x G1G2 | |
| --- | --- | --- | --- | --- | --- | --- | --- | --- | --- | --- | --- | --- | --- | --- | --- | --- | --- | --- |
| Phenotypic traits |  | F_dF_ | P^b^ |  | F_dF_ | P^b^ |  | F_dF_ | P^b^ |  | F_dF_ | P^b^ |  | F_dF_ | P^b^ |  | F_dF_ | P^b^ |
| Rosette diameter 3 weeks |  | **10.705_2,236_** | **<0.001** |  | **13.361_1,24_** | **0.003** |  | 0.081_1,236_ | 0.905 |  | 0.568_2,236_ | 0.795 |  | 0.098_2,236_ | 0.907 |  | 0.522_1,236_ | 0.795 |
| Rosette leaves FFD |  | **63.467_2,236_** | **<0.001** |  | **20.285_1,24_** | **<0.001** |  | 0.105_1,236_ | 0.746 |  | 1.571_2,236_ | 0.245 |  | 2.432_2,236_ | 0.126 |  | 4.335_1,236_ | 0.067 |
| Height |  | **156.093_2,236_** | **<0.001** |  | **86.038_1,24_** | **<0.001** |  | **5.202_1,236_** | **0.041** |  | 3.105_2,236_ | 0.065 |  | 1.830_2,236_ | 0.190 |  | 0.215_1,236_ | 0.643 |
| Siliques per branch |  | **16.803_2,236_** | **<0.001** |  | 0.429_1,24_ | 0.519 |  | 1.690_1,236_ | 0.341 |  | 0.994_2,236_ | 0.436 |  | 0.989_2,236_ | 0.436 |  | 2.007_1,236_ | 0.341 |
| Total branches |  | **4.582_2,236_** | **0.026** |  | 45.254_1,24_ | **<0.001** |  | 0.018_1,236_ | 0.944 |  | 1.084_2,236_ | 0.595 |  | 0.223_2,236_ | 0.944 |  | 0.005_1,236_ | 0.944 |

^b^P-values were adjusted for multiple testing according to Benjamini and Hochberg (1995)
