# Supplementary material for: Phenotypic Effects of Salt and Heat Stress over Three Generations in Arabidopsis thaliana
Source: PLoS One. 2013 Nov 14;8(11):e80819. doi: 10.1371/journal.pone.0080819 (PMC3828257; doi:10.1371/journal.pone.0080819)
Supplement: Table S9 — Effect of two generations of heat treatment (G1 and G2) vs. two generations of control treatment on variances of traits measured under G3 heat and control conditions. (DOCX) [file pone.0080819.s010.docx]

**Table S7**: Effect of two generations of heat treatment (G1 and G2) vs two generations of control treatment on trait variances under G3 heat and control conditions, separately for each genotype.

^b^P values were adjusted for multiple testing according to Benjamini and Hochberg (1995)

***: P value < 0.001; **: P value < 0.01; *: P value < 0.05; ·: P value < 0.1
